# Phosphomimetic Tyrosine Mutations in Spa47 Inhibit Type Three Secretion ATPase Activity and *Shigella* Virulence Phenotype

**DOI:** 10.3390/pathogens11020202

**Published:** 2022-02-03

**Authors:** Koleton D. Hardy, Nicholas E. Dickenson

**Affiliations:** Department of Chemistry and Biochemistry, Utah State University, Logan, UT 84322, USA; koleton.hardy@usu.edu

**Keywords:** *Shigella*, pathogen, phosphomimetic, T3SS, regulation, tyrosine, ATPase, PTM, injectisome, phosphorylation

## Abstract

*Shigella* is a highly infectious human pathogen responsible for 269 million infections and 200,000 deaths per year. *Shigella* virulence is absolutely reliant on the injection of effector proteins into the host cell cytoplasm via its type three secretion system (T3SS). The protein Spa47 is a T3SS ATPase whose activity is essential for the proper function of the *Shigella* T3SS needle-like apparatus through which effectors are secreted. A phosphoproteomics study recently found several *Shigella* T3SS proteins, including Spa47, to be tyrosine phosphorylated, suggesting a means of regulating Spa47 enzymatic activity, T3SS function, and overall *Shigella* virulence. The work presented here employs phosphomimetic mutations in Spa47 to probe the effects of phosphorylation at these targeted tyrosines through in vitro radiometric ATPase assays and circular dichroism as well as in vivo characterization of T3SS secretion activity, erythrocyte hemolysis, and cellular invasion. Results presented here demonstrate a direct correlation between Spa47 tyrosine phosphorylation state, Spa47 ATPase activity, T3SS function, and *Shigella* virulence. Together, these findings provide a strong foundation that leads the way to uncovering the specific pathway(s) that *Shigella* employ to mitigate wasteful ATP hydrolysis and effector protein secretion when not required as well as T3SS activation in preparation for host infection and immune evasion.

## 1. Introduction

*Shigella* are Gram-negative, rod-shaped, facultative anaerobic bacteria that are closely related to *Escherichia coli* and belong to the large family *Enterobacteriaceae*. Each of the four species of *Shigella* are human pathogens that are most commonly spread through the fecal–oral route and contaminated water supplies [1]. Exposure to as few as 10–100 organisms supports the onset of a full shigellosis infection resulting in a severe and potentially deadly form of bacillary dysentery [2]. In fact, *Shigella* is responsible for an estimated 269 million infections worldwide and as many as 200,000 deaths annually [3]. While shigellosis infections disproportionately burden developing regions of the world where there is limited access to clean drinking water and antibiotic treatment, there is also an urgent concern surrounding the rapid emergence of antibiotic-resistant *Shigella* strains in both developing and industrialized nations throughout the world [4,5]. These recent developments underscore the need to better understand the mechanism(s) that *Shigella* uses to infect human hosts and identify potential targets for the formulation of efficient non-antibiotic countermeasures against shigellosis.

Like many Gram-negative pathogenic bacteria, *Shigella* rely absolutely on a type three secretion system (T3SS) for virulence [6,7,8,9]. The *Shigella* T3SS injects bacterial effector proteins directly into the cytoplasm of eukaryotic host cells, subverting a milieu of host cell functions and allowing the bacteria to invade the cells of the colonic epithelium, evade host immune defenses, and spread throughout the infected tissues [10,11]. Structurally, the T3SS injectisome responsible for these processes resembles a hypodermic syringe and needle comprised of several distinct regions that are highly structurally conserved among the pathogens that utilize them [12,13,14]. The cytoplasmic bulb of the injectisome resides within the cytoplasm of the bacteria and is often referred to as the sorting platform as recent structural and biochemical studies have shown that it contains multiple copies of a T3SS ATPase necessary for the proper function of the system, regulatory proteins capable of enhancing and inhibiting activity of the ATPase, and T3SS chaperone binding proteins that presumably guide protein secretion through the apparatus by recognizing and removing T3SS chaperones from secreted translocator proteins [15,16,17,18]. The basal body of the apparatus interacts with the cytoplasmic bulb and provides an anchor for the apparatus that spans the inner and outer membranes of the bacteria. This region has been suggested to contain protein complexes that provide dedicated channels that utilize the potential energy of proton and other ion-derived electrochemical gradients across the inner membrane to support protein secretion through the apparatus [19,20,21]. The hallmark needle-like structure itself is a homo-oligomeric complex that extends approximately 45 Å–80 Å from the basal body and terminates slightly beyond the associated lipopolysaccharide layer, providing a ~2.5 Å (inner diameter) unidirectional conduit that guides the secretion of T3SS effectors from the bacterial cytoplasm [22,23]. Finally, the distal tip of the needle is terminated with a multi-protein tip complex that acts as an environmental sensor, prevents premature protein secretion through the apparatus, and inserts a translocon pore in the eukaryotic host cell membrane, providing direct access for T3SS protein secretion into the host cell cytoplasm [24,25,26,27,28,29].

The clear correlation between T3SS activity and *Shigella* virulence has fueled a number of studies defining the regulatory means that *Shigella* utilize to prevent wasteful expression and secretion of T3SS proteins prior to encountering a host’s colonic epithelial cells. These regulatory mechanisms include the temperature-dependent transcriptional regulation of critical T3SS components and both bile salt and lipid sensation by proteins within the tip complex that trigger subsequent steps in protein secretion and apparatus maturation [25,26,30,31,32,33]. More recently, a pioneering tyrosine phosphoproteomics study in *Shigella* found that approximately 15% of *Shigella* proteins contained one or more posttranslational tyrosine phosphorylation modifications [34]. While the *Shigella* T3SS ATPase, Spa47, represents only one of the ≥573 proteins identified with phosphotyrosine modifications in the proteome, Spa47’s recently described role(s) in supporting T3SS injectisome formation, protein secretion, and overall pathogen virulence suggest that posttranslational phosphorylation may serve as a novel and potent means for regulating T3SS activity and *Shigella* virulence [35,36], leading to the question of whether these modifications are an integral part of an intricate regulatory process. 

Here, we utilize a series of phosphomimetic substitutions at two tyrosine residues identified as targets for in vivo phosphorylation. In vitro biophysical characterization of the purified mutants together with enzyme kinetic analyses of both isolated monomeric and oligomeric Spa47 mutant species provide insight into the importance of each tyrosine in Spa47 structure, stability, and enzymatic function and inform on the potential impact of the posttranslational phosphorylation of each. Subsequent in vivo characterization of *Shigella* strains expressing each of the engineered Spa47 mutants further uncover a strong virulence dependence on mutations to the highly conserved Spa47^Y274^ target. Together, the findings presented here clearly demonstrate the importance of the identified Spa47 tyrosine residues in both Spa47 function and *Shigella* virulence, providing a strong foundation for follow-up studies uncovering the specific regulatory pathway(s) responsible for Spa47 regulation as well as identifying a priority target for the development of much needed non-antibiotic T3SS ATPase inhibitors.

## 2. Results

### 2.1. Structural Insights into the Roles of Post-Translationally Targeted Tyrosines in Spa47

**Figure 1:**
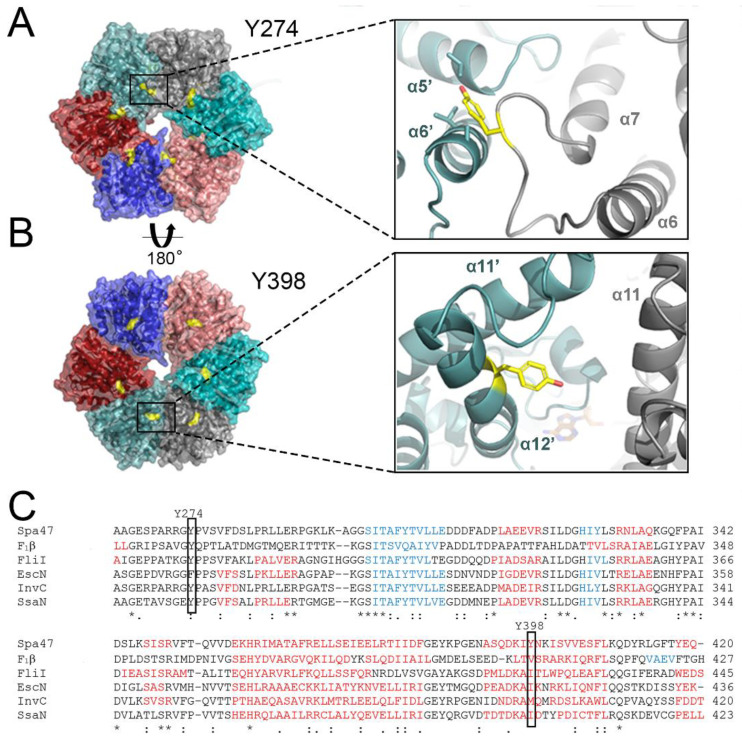
A homo-hexameric Spa47 model illustrating the molecular environments of phosphorylation targeted tyrosines. (**A**) Spa47^K165A^ (PDB: 5SYP) was modeled as an activated homo-hexamer, based on the hexameric F1 ATP synthase structure (PDB: 1BMF). Each Spa47 monomer subunit is colored independently, Y274 is illustrated in yellow. The insert provides a closer view of the protomer interface involving Y274. Referenced helices are labeled in each protomer, with the prime (‘) designation differentiating the two protomers. (**B**) The structure is rotated up 180 degrees with Y398 illustrated in yellow on the cyan protomer. The insert provides a closer view of the location of Y398 within the protomer interface. (**C**) Sequence alignment of ATPases from several organisms was performed using Clustal Omega. Fully conserved residues (*), residues with strongly similar properties (:), and residues with weakly similar properties (.) are identified. Secondary structure predictions were generated using PSIPRED with alpha helices colored in red and beta strands in blue. Residues aligning to the *Shigella* Spa47 tyrosines 274 and 398 are identified using boxes. Uniprot accession numbers used for sequence alignment are Q6XVW8, P00829, P26465, Q7DB71, B5RDL8, P74857, for Spa47, F1 ATPase B subunit, InvC, YscN, EscN, SsaN, respectively.

To better understand the potential effects of phosphorylating tyrosines 274 and 398, a homo-hexameric Spa47 model was generated as described previously [35]. Briefly, the recently solved 2.15 Å structure of Spa47^K165A^ (PDB ID 5SYP) was aligned to each of the protomers of the 2.8 Å hetero-hexameric F1 α_3_β_3_ ATP synthase structure (PDB ID 1BMF) using PyMOL. The location of the tyrosine residues with respect to the interfacial nucleotide binding site was considered to determine whether phosphorylation would likely lead to direct effects within the active site of Spa47. As seen in Figure 1, the tyrosine residues are unlikely to participate directly in catalysis as they are located on opposite poles of the complex, nearly 50 Å from one another, and each is greater than 20 Å from the active site that is positioned within a protomer interface approximately midway between the highlighted tyrosines. Specifically, tyrosine 274 is located on a long loop connecting helices α6 and α7 (Figure 1A). From its position on this loop at the protomer interface, tyrosine 274 can potentially interact with residues displayed on helices α5 and α6 of the adjacent protomer, likely including hydrophobic interactions between the phenyl ring of Y274 and the aliphatic sidechains of V221 and/or V261 (Figure 1A). Tyrosine 398, on the other hand, is located on the opposite pole of the Spa47 structure relative to Y274. A 180° rotation of the Sp47 model shows that Y398 is also located at a protomer interface within the homo-hexameric model but is located within helix α12, limiting local flexibility relative to the loop-bound Y274. Additionally, the model suggests that from this position, Y398 is too far from the adjacent protomer to support either electrostatic or hydrophobic interaction (Figure 1B). Previous identification of in vivo phosphorylation at both of these tyrosine residues [34], together with their putative location at catalytically-critical Spa47 interfaces [37] suggests that phosphorylation could provide a means for regulating Spa47 activity, T3SS function, and ultimately *Shigella* virulence. While Spa47 currently represents the only T3SS ATPase for which posttranslational phosphorylation has been uncovered, it is tempting to speculate that such a regulatory mechanism would be conserved among T3SS ATPases from different pathogens. In fact, a Clustal Omega sequence alignment of Spa47, F_1_β, and several related T3SS ATPases identifies a near perfect conservation of Y274 with the only non-identical residue in the alignment being a phenylalanine in EscN from *E. coli,* providing anecdotal support for the importance of Y274 in T3SS ATPase function and its potential as a regulatory target (Figure 1C). Evaluation of the equivalent positions to Y398 in the isozymes, on the other hand, shows a stark lack of conservation, with none of the other aligned ATPase sequences containing a tyrosine (or any other residue capable of being phosphorylated) at this position, suggesting that it is not involved in a conserved mechanistic or regulatory role, though not necessarily ruling out its importance in the *Shigella* T3SS.

### 2.2. Phosphomimetic Tyrosine Mutations Impact Spa47 ATPase Activity

**Figure 2:**
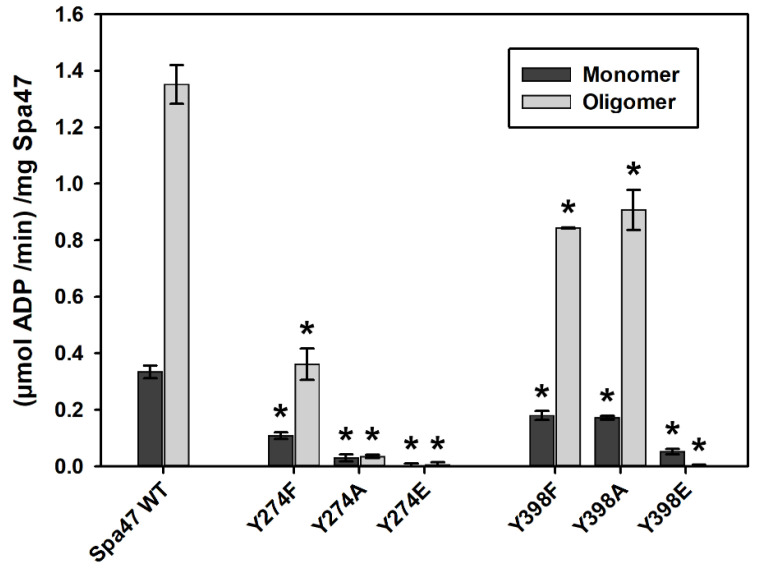
Kinetic analysis of the Spa47 tyrosine mutants engineered in this study. Isolated wild-type Spa47 monomers and oligomers both hydrolyze ATP, though the oligomer (grey bars) displays an enhanced rate of hydrolysis compared to monomeric Spa47 (black bars). Activity was additionally evaluated for both monomeric and oligomeric forms of each Spa47 tyrosine mutant in this study. Each data set represents the mean rate of hydrolysis ± S.D. of three independent kinetic experiments spanning two independent protein preparations. (*) Indicates a statistical significance compared to the appropriate oligomer state of wild-type Spa47 (one-way ANOVA followed by a Dunnett’s post hoc test, *p* ≤ 0.05).

We have previously shown that stable Spa47 oligomers display significantly enhanced ATPase activity compared to isolated monomeric Spa47 as oligomerization supports critical active site contributions from adjacent protomers [35,36]. Furthermore, disruption of interfacial salt bridges along the protomer interface was shown to be detrimental to catalytic activity in vitro and T3SS function in vivo, supporting the idea that disrupting interactions with even a single residue can reduce/eliminate Spa47 activity [37]. The potential effects of phosphorylating Y274 and Y398 on Spa47 ATPase activity were tested by independently mutating each of them to the commonly utilized tyrosine phosphomimetic glutamate as it introduces a negative charge in the “R” group and provides similar electrostatic properties as a phosphorylated tyrosine [38,39]. The native tyrosine residues were additionally mutated to phenylalanine as this mutation maintains the aromatic “R” group via the phenyl ring while eliminating the alcohol functional group required as a nucleophile that allows posttranslational phosphorylation. Finally, tyrosine to alanine mutations were engineered to eliminate the “bulk” of the native sidechain, the ability to participate in intermolecular salt bridges, and the ability to become phosphorylated. The effect of each of the engineered Spa47 point mutations on ATPase activity was determined for isolated monomeric and oligomeric Spa47 using a radiometric α-^32^P-ATP multiple time point activity assay (Figure 2). The in vitro activity results show that the full-length wild-type monomeric and oligomeric Spa47 species behaved as previously reported, hydrolyzing ATP at rates of 0.33 ± 0.02 (μmol ADP/min)/mg Spa47 and 1.35 ± 0.07 (μmol ADP/min)/mg Spa47, respectively [40]. Mutations to Y274 show a clear pattern in which the extent of reduction in ATPase activity of both the monomeric and oligomeric species increases moving from the native tyrosine to phenylalanine, to alanine, and to glutamate, with the phosphomimetic glutamate mutation resulting in complete elimination of activity (0.00 ± 0.01 μmol ADP/min)/mg Spa47 and 0.01 ± 0.01 μmolADP/min)/mg Spa47 for the monomeric and oligomeric conditions, respectively). 

The Y398E mutant exhibited similar effects with the monomeric and oligomeric fractions, hydrolyzing ATP at negligible rates of 0.05 ± 0.01 (μmol ADP/min)/mg Spa47 and −0.02 ± 0.03 (μmol ADP/min)/mg Spa47, supporting the idea that phosphorylation of either tested tyrosine would inhibit Spa47 activity. Interestingly, however, the engineered Y398F and Y398A mutants displayed equivalent and modest reductions in Spa47 activity, suggesting that contrary to Y274, a significant charge perturbation is required at this position to provide an appreciable reduction in activity and that the large hydrophobic phenyl sidechain of the phenylalanine does not provide an advantage to activity over the small alanine methyl group in this position. 

The observed effects on Spa47 activity resulting from independently mutating Y274 and Y398 led us to consider the possibility that tyrosine 274 and tyrosine 398 may both be phosphorylated on a single Spa47 protein as a means of even further enhancing the inhibitory effects on Spa47. Unfortunately, the phosphotyrosine proteomics study that guided the identification of these tyrosines as targets was not sensitive to this possibility as the two sites are located 124 residues apart and the proteolytic digestion employed prior to mass spectrometry analysis prevented detection of the dual-phosphorylated product in question [34]. We attempted to address this possibility by engineering a Spa47^Y274E/Y398E^ double mutant with both tyrosines mutated to phosphomimetic glutamates. Despite significant efforts, however, recombinant expression of the double mutant resulted in insoluble inclusion bodies of the engineered Spa47^Y274E/Y398E^ product. While this does not necessarily rule out the possibility of Spa47 phosphorylation at multiple sites, it prevented us from further examining the Spa47^Y274E/Y398E^ mutant in this study and suggests that either of the singly phosphorylated products is more stable and a more likely means of T3SS regulation in vivo.

### 2.3. Phosphomimetic Tyrosine Mutations Impact Spa47 Thermal Stability 

Far-UV circular dichroism (CD) was employed to determine whether the engineered mutations result in perturbations to the overall protein secondary structure and/or overall structural stability of Spa47. Far-UV circular dichroism spectra and thermal unfolding profiles were collected and evaluated for both monomeric and oligomeric isolates of the Spa47 tyrosine mutants (Supplementary Figure S1). CD data were not collected for the Spa47^Y398E^ oligomer species as limited stability/solubility at concentrations required for CD analysis prevented its characterization. Each of the collected spectra are characteristic of proteins containing significant alpha helical character (consistent with previously published CD spectra and crystal structures of Spa47) [35] and none are indicative of proteins with significantly compromised secondary structures. In addition to similar spectral characteristics, the observed wild-type Spa47 melt temperatures of 44.4 ± 0.2 °C and 43.5 ± 1.5 °C for the monomeric and oligomeric species, respectively, are in good agreement with those determined previously (Table 1) [16]. The mutations generally had little effect on the melt temperatures of the isolated oligomers, with only the Y398A mutant displaying a significantly altered Tm (40.5 ± 1.9 °C). The thermal stability of the monomeric species, however, appears to be more sensitive to the mutations than the isolated oligomers. In fact, with the exception of the Y398F mutant, each exhibited significantly decreased melt temperatures compared to WT Spa47. The Y398E mutation resulted in the largest decrease in stability with a Tm of only 36.0 ± 0.6 °C, with similar, but perhaps enhanced, effects on the mutant’s structural stability likely preventing characterization of the corresponding mutant oligomeric species.

### 2.4. Phosphomimetic Substitutions to Spa47^Y274^, but Not Spa47^Y398^, Regulate Protein Secretion through the T3SS Injectisome 

**Figure 3:**
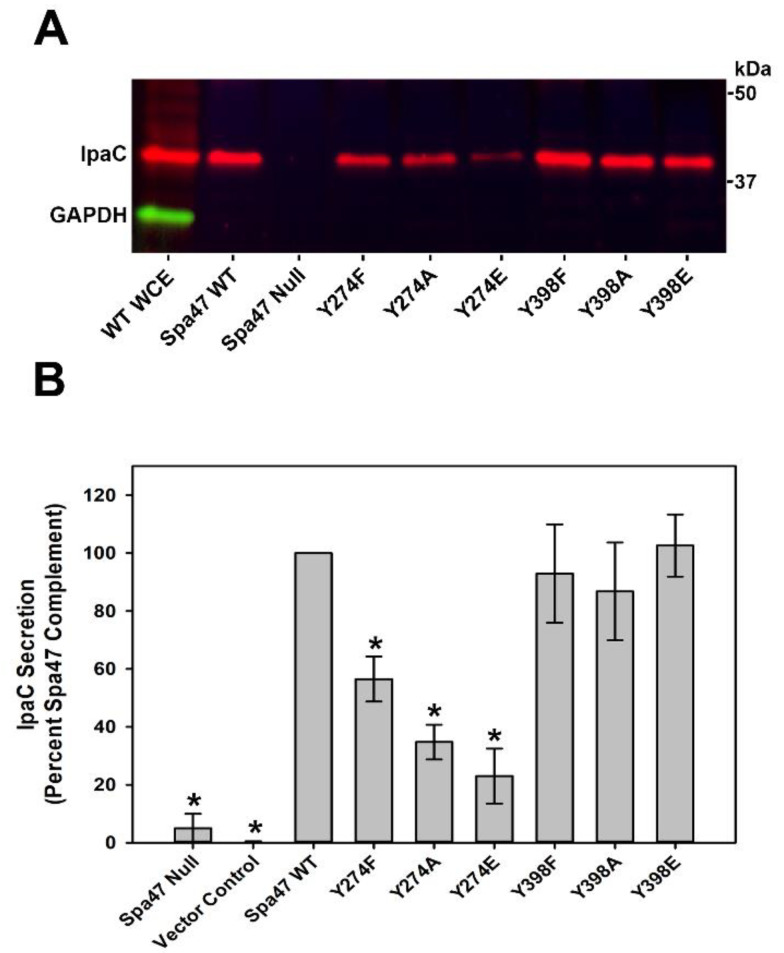
Immunoblot analysis of Congo Red-induced IpaC secretion profiles for *Shigella* strains expressing the engineered Spa47 mutants. (**A**) Representative immunoblot of actively secreted IpaC by the tested *Shigella* mutants. The cytoplasmic enzyme GAPDH serves as a cytoplasmic control validating that the assay is specifically sensitive to secreted proteins as it is observed in the whole cell extracts (WCE) but not in the supernatant containing the secreted IpaC protein. (**B**) The levels of secreted IpaC are quantified via intensity and reported relative to a *S. flexneri* strain expressing wild-type Spa47 (set to 100%). The reported values represent the mean levels ± S.D. from three independent analyses spanning two biological replicates. (*) Indicates statistical significance compared to the wild-type Spa47 strain (one-way ANOVA followed by a Dunnett’s post hoc test, *p* ≤ 0.05).

Spa47-catalyzed ATP hydrolysis is essential for protein secretion through the T3SS needle-like injectisome and is directly associated with *Shigella* virulence. To determine the effect that the tested phosphomimetic Spa47 mutations had on the ability of *Shigella* to actively secrete effector proteins through the injectisome, levels of the secreted *Shigella* translocator protein IpaC were quantified following the Congo Red activation of *Shigella* strains expressing each of the engineered Spa47 tyrosine mutants (Figure 3). While none of the tested mutations targeting the non-conserved Y398 resulted in a change in secretion levels of the translocator protein IpaC, mutations to the highly conserved Y274 produced a distinct pattern in which the secretion levels decrease moving from the native tyrosine to the most conserved mutation of phenylalanine, to alanine, and finally to the phosphotyrosine mimic glutamate. The Spa47^Y274E^ mutant reduced IpaC secretion by 77 ± 9%, suggesting that this mutation not only eliminates Spa47 catalysis in vitro (Figure 2), but also significantly inhibits T3SS function in vivo. Previous work identifying posttranslational phosphorylation of Y274 in vivo [34], together with these findings, strongly suggest that posttranslational phosphorylation of Y274 could influence T3SS activity and provide a non-transcriptionally derived means for regulating T3SS activity in vivo.

### 2.5. Spa47^Y274^, but Not Spa47^Y398^, Is Critical to Shigella Virulence Phenotype

Secretion of the translocator protein IpaC through the injectisome and its incorporation into the translocon pore in the host cell membrane marks the final step in the maturation of the type three secretion apparatus and ultimately allows the secretion of effector proteins into the eukaryotic host cell cytoplasm [15,25]. Secretion of T3SS effectors into the host cytoplasm then supports invasion of the host cell, escape from the resulting vacuole, and evasion of host immune responses [6,7,8,9,10,11]. *Shigella* strains expressing the engineered Spa47 phosphomimetics resulted in significantly different effects on IpaC secretion levels, specifically suggesting that phosphorylation of Spa47^Y274^ inhibits protein secretion while phosphorylation of Y398 does not (Figure 3). To better understand the consequences that Spa47 phosphorylation has on T3SS-mediated *Shigella* virulence, *Shigella* strains expressing the engineered phosphomimetic Spa47 tyrosine mutants were examined for their ability to both lyse red blood cells (RBC) and to invade cultured epithelial cells (Table 2). The RBC hemolysis assay is specifically sensitive to the pathogen’s ability to form the T3SS translocon pore within a host cell membrane, a critical first step in *Shigella*’s virulence pathway that is known to be directly related to secretion of IpaC through the injectisome and be absolutely dependent on enzymatically-competent Spa47 [24,36,41,42]. The hemolysis results correlate strongly with the IpaC secretion data also collected in this study (Figure 2 and Figure 3) with the hemolytic phenotype of the Spa47^Y274F^ strain essentially equivalent to the wild-type *Shigella* strain and the Spa47^Y274A^ and phosphomimetic Spa47^Y274E^ strains exhibiting significantly reduced hemolytic phenotypes at 78 ± 8% and 46 ± 3% of wild-type, respectively. Again, consistent with the collected IpaC secretion data, none of the mutants expressing Y398 variants exhibited reduced hemolysis phenotypes, and rather, each resulted in enhanced hemolytic phenotype, though the origin of this enhancement remains enigmatic.

Like RBC hemolysis, the ability of *Shigella* to invade eukaryotic epithelial cells also requires the formation of T3SS translocon pores in the host cell membrane, but additionally requires the active secretion of several effector proteins into the host cell cytoplasm to subvert critical cellular functions such as actin polymerization and induce macropinocytotic uptake of the bacteria, testing the full range of function of the T3SS with respect to *Shigella*’s ability to gain access to host cell cytoplasm. In a similar, but exaggerated trend, the *Shigella* strain expressing the conservative Spa47^Y274F^ mutant was not affected with respect to invasion phenotype, while the strains expressing the Spa47^Y274A^ and phosphomimetic Spa47^Y274E^ mutants demonstrated significantly reduced invasion phenotypes at 51 ± 9% and 30 ± 11% of wild type, respectively (Table 2). Again, consistent with the other phenotype studies presented here, however, none of the Spa47^Y398^ mutants significantly affected invasion phenotype. Together, these findings demonstrate the importance of the highly conserved Spa47^Y274^ residue in supporting overall *Shigella* virulence with the Spa47^Y274E^ mutation mimicking the phosphorylation of the native tyrosine resulting in significant defects in translocator protein secretion through the T3SS injectisome and significantly reduced hemolytic and invasion phenotypes. Ultimately, these data support the hypothesis that the posttranslational phosphorylation of Spa47^Y274^ may serve as a critical native means of regulating *Shigella* T3SS activity and virulence as well as identifying a potentially potent target for non-antibiotic small molecule therapeutics against not only *Shigella*, but perhaps the many pathogens that rely on T3SSs as critical virulence factors and share this conserved tyrosine residue in their T3SS-associated ATPases.

## 3. Discussion

Some of the first descriptions of bacterial T3SSs (not including flagellar T3SSs) were those of *Pseudomonas* and *Yersinia* in 1993, providing important insight into their virulence mechanisms [43,44]. Two years later, Sansonetti and colleagues made the important discovery of a single T3SS in *Shigella*, initiating a new direction of study into the mode of infection of yet another important human pathogen [45]. Today, more than twenty bacterial species are known to express T3SSs as critical components in their virulence arsenals [8], and work continues to dissect the structure, function, and regulatory mechanisms of the T3SSs in each of the organisms that express them. Like many of the pathogens expressing T3SSs, *Shigella* virulence is absolutely reliant on a fully functional T3SS capable of appropriately timing the injection of effector proteins into the cytoplasm of host cells [6,7,8,9]. A great deal of work has been done to understand the transcriptional regulation of T3SS gene expression in which invasion genes located on the 220kb invasion plasmid are controlled by the transcriptional regulator VirB, whose expression is in turn controlled by the temperature-dependent expression of the master regulator VirF [30,31,32]. As a human enteric pathogen, initiating transcription of VirB at temperatures exceeding 30 °C prevents the unnecessary and wasteful expression of *Shigella* T3SS genes while outside of the host organism. While *Shigella* clearly rely on transcriptional control to regulate virulence and arm its T3SS arsenal once ingested by the host, specific targeting of the colonic epithelium and other carefully orchestrated events such as vacuolar escape and induced apoptosis of macrophages make it highly unlikely that these early transcriptional regulation events alone can support the strict timing required during *Shigella* infection. 

Most likely, the well-described transcriptional regulation of T3SS genes is complemented by additional, faster acting, regulatory processes sensitive to specific environmental cues. For example, from their positions at the distal tip of the injectisome, the proteins IpaD and IpaB each trigger critical steps in T3SS injectisome tip maturation following exposure to the bile salt deoxycholate and host membrane components, respectively, preparing *Shigella* for invasion of the colonic epithelium [25,26,46,47,48,49]. Furthermore, we have recently solved a series of high-resolution structures of the *Shigella* T3SS protein, Spa47, and have provided enzymatic characterization identifying it is an oligomerization-activated ATPase essential for T3SS function and *Shigella* virulence [35,36,40]. Its role in supporting both proper injectisome formation and protein effector secretion makes Spa47 an attractive target for native and therapeutic regulation/inhibition of T3SS activity. In fact, a recent study confirmed this when the T3SS protein MxiN was identified as a Spa47 chaperone, differentially regulating ATPase activity dependent on the oligomeric state of Spa47, suggesting that MxiN is a critical component in the regulatory pathway controlling *Shigella* T3SS activity [16]. Follow-up works targeting Spa47’s role in the *Shigella* T3SS identified several non-competitive small molecule Spa47 inhibitors that not only inhibit ATPase activity in vitro, but efficiently reduce in vivo T3SS protein secretion by as much as 95%, confirming the potential for Spa47 as a regulatory target and providing a strong foundation for the development of much needed non-antibiotic therapeutics against *Shigella* infections [50,51]. 

Standish and coworkers recently completed a tyrosine phosphoproteomics study for *Shigella flexneri*, uncovering 905 unique tyrosine posttranslational phosphorylation sites that spanned ≥573 proteins [34]. Twenty-one of the identified proteins were T3SS proteins, providing the first direct evidence of *Shigella* T3SS protein posttranslational phosphorylation. While it is likely that these modifications play important regulatory roles in many of the identified protein targets, this specifically opened a new avenue of opportunity for exploring the role of posttranslational modification in regulating *Shigella* virulence through T3SS activity. In line with this reasoning, three of the T3SS proteins found to contain posttranslational phosphotyrosine modifications include MxiN, VirB, and Spa47. MxiN has been shown to interact with Spa47 and differentially regulate ATPase activity of monomeric and oligomeric forms of the enzyme [16] and plaquing and qualitative secretion assays of phosphomimetic mutants of the latter two [34] support the idea that phosphorylation of these proteins may reduce *Shigella* virulence phenotype. Interestingly, this study showed that knocking out Wzc, the only known tyrosine kinase in *Shigella*, had no effect on the tested phenotype, suggesting that at least one yet-to-be identified tyrosine kinase/phosphatase pair is responsible for controlling the phosphorylation state of *Shigella* T3SS proteins, reminding us of just how little is currently known about T3SS regulation.

In this study, we leveraged the ability to express and purify enzymatically active Spa47 to examine the effects of the identified phosphorylation events on Spa47 ATPase activity and its role in *Shigella* T3SS function and pathogen virulence. Several mutations were made to each of the tyrosine residues identified previously as targets for phosphorylation (Y274 and Y398) and all resulted in significant decreases in ATPase activity by Spa47, with activity essentially abolished in the phosphomimetic glutamate substitution mutants, clearly demonstrating that both tyrosine residues are essential for appropriate activity in vitro. Although the specific mechanism for this disruption is not entirely clear, similar mutations made to interfacial amino acids in Spa47 have been linked to disruption of a complex intermolecular hydrogen bonding network that ultimately affects the geometry of critical residues within the interfacial active site of the complex [37]. While distant from the active site, both Y274 and Y398 are also located at the interface of Spa47 protomers in the context of the activated Spa47 oligomer and perhaps they too are involved in intermolecular interaction that support an ATPase competent structure. Work is currently underway to solve X-ray crystal structures of the mutants engineered in this study and the findings will likely shed additional light on the specific structural role(s) of these important tyrosines. 

Extensive in vivo phenotype studies including induced T3SS translocator secretion, eukaryotic host cell invasion, and erythrocyte hemolysis assays were additionally performed in this study to assess the effect of the engineered mutations of tyrosines 274 and 398 on T3SS function and *Shigella* virulence. While each assay assesses specific aspects of T3SS function and different steps in the infection mechanism, each of these functions is absolutely reliant on proper Spa47 ATPase activity, providing an important readout with respect to the potential effects of phosphorylation at the identified tyrosine targets. Somewhat surprisingly, we found that despite clear reductions in ATPase activity of the isolated Spa47^Y398^ mutants, none of the *Shigella* strains expressing mutations to tyrosine 398 exhibited reduced T3SS function or *Shigella* virulence (in fact all resulted in a modestly enhanced hemolysis phenotype compared to wild-type), as perhaps the deleterious effects of the mutation were overcome by favorable protein interactions in the context of the assembled T3SS injectisome, making it likely that phosphorylation observed at Y398 in the phosphotyrosine proteomics study [34] is the result of an enigmatic and promiscuous kinase and not one that drives regulation of T3SS activity. The *Shigella* strains expressing the engineered Spa47^Y274^ mutants, however, resulted in a consistent pattern of reducing virulence phenotype as the mutation changed from phenylalanine to alanine to glutamate, as would be expected if phosphorylation of Y274 reduced T3SS function through a reduction in Spa47 activity. Furthermore, it is tempting to speculate that the highly conserved placement of tyrosine in the position equivalent to Spa47^Y274^ in homologous T3SS ATPases is the result of a conserved regulatory pathway that takes advantage of this virulence modulation in related pathogens. Little is currently known, however, regarding the impact/use of posttranslational modifications in controlling type three secretion system ATPase activity and it is hopeful that these studies will provide the momentum necessary for follow-up work that not only uncovers the molecular details of the observed effects in *Shigella*, but that also extend these studies to explore the potential for a conserved pathway in other T3SS-expressing pathogens. 

In conclusion, the data presented here provide the first enzymatic study of a PTM on a T3SS ATPase and correlate these findings to *Shigella* T3SS function and virulence. Taken together, the presented in vitro enzyme characterization and in vivo *Shigella* virulence phenotype characterizations strongly support the hypothesis that the posttranslational phosphorylation of Spa47^Y274^ reduces Spa47 activity, T3SS function, and *Shigella* virulence. The next critical steps in continuing this work include identifying the specific kinase(s) and phosphatase(s) involved in the Spa47 phosphorylation pathway and ultimately describing the stimuli and mechanisms that regulate their action. Uncovering these pathways not only provides critical information that helps to close the significant gap in our understanding of how T3SSs are regulated, but also identifies a potentially potent target for the development of non-antibiotic therapeutics against infections by *Shigella* and related bacterial pathogens.

## 4. Materials and Methods

### 4.1. Materials

The Spa47 null *Shigella flexneri* strain was generated and validated previously by the Allaoui lab [52]. Rabbit polyclonal anti-IpaC antibodies and the pWPsf4 expression plasmid were generous gifts from Wendy Picking at the University of Kansas and have been validated previously [35,42]. *E. coli* NovaBlue and Tuner (DE3) cells and ligation mix were purchased from Novagen (Madison, WI, USA). The pTYB21 plasmid, PCR buffer, Phusion polymerase, chitin affinity resin, and restriction enzymes were from New England Biolabs (Ipswich, MA, USA). The synthetic Spa47 gene and PCR primers were from Integrated DNA Technologies (Coralville, IA, USA). All antibiotics, IPTG, and DTT were purchased from Gold Biotechnology (St. Louis, MO, USA). The anion exchange and size exclusion columns were from Cytiva (Marlborough, MA, USA). ATP was from Sigma–Aldrich (St. Louis, MO, USA) and radioactive α-^32^P-ATP was from Perkin Elmer (Boston, MA, USA). All other solutions and chemicals were of reagent grade.

### 4.2. Cloning

The wild-type sequence gene for Spa47 was purchased from Integrated DNA Technologies and ligated into the commercial expression plasmid pTYB21, which encodes an N-terminal chitin binding domain (CBD) and cleavable intein linker. It was then heat shock transformed into *E. coli* NovaBlue cells, and resulting colonies were screened for *spa47* and sequence-verified by Genewiz (South Plainfield, NJ, USA). The wild-type Spa47 gene was also cloned into the high copy number, pUC18-based pWPsf4 plasmid [42] for constitutive expression in *Shigella*. *Shigella* strains expressing wild-type and mutant Spa47 from the pWPsf4 plasmid were generated by electroporation of a *S. flexneri spa47* knockout strain. The Spa47 point mutants in this study were made in both pTYB21 and pWPsf4 backbones, used inverse PCR, and were transformed into *E. coli* and *S. flexneri*, respectively, following sequence validation.

### 4.3. Protein Expression and Purification

*E. coli* Tuner (DE3) cells containing the Spa47 genes in pTYB21 were grown to saturation in LB Broth (Miller) supplemented with 0.1 mg/mL ampicillin. A small amount of the saturated culture was used to inoculate one-liter cultures in Terrific Broth media again supplemented with 0.1 mg/mL ampicillin. The cultures were grown in a shaker at 37 °C and 200 RPM to an OD600 of 0.8 prior to cooling to 17 °C and induction with 1 mM IPTG for 20 h at 17 °C and 200 RPM. All subsequent protein purification steps were performed at 4 °C. Following expression, cells were pelleted by centrifugation, resuspended in chitin binding buffer (20 mM Tris, 500 mM NaCl, 0.2 mM AEBSF. pH 7.9), and lysed by sonication. The sonicated product was centrifuged and the supernatant containing soluble protein run over a chitin affinity column. The chitin resin was then rinsed generously with chitin binding buffer followed by overnight incubation in intein cleavage buffer (20 mM Tris, 500 mM NaCl, 50 mM DTT, pH 7.9) to cleave Spa47 from the chitin binding domain (CBD). Several protein elutions were collected and assessed via SDS-PAGE. The elutions were combined and adjusted to a final concentration of 20 mM Tris, 100 mM NaCl, 5 mM DTT, pH 7.9, and further purified via negative selection using a Q Sepharose FF anion exchange column. The purified protein was concentrated using centrifugal filter units with 30-kDa molecular weight cut offs and further purified/characterized using a Superdex 10/300 size exclusion column equilibrated with 20 mM Tris, 100 mM NaCl, 5 mM DTT, pH 7.9. Spa47 concentrations were determined using SDS-PAGE in-gel densitometry with bovine serum albumin as a standard, as validated previously [35]. All Spa47 concentrations are reported in monomer concentration units for consistency and clarity.

### 4.4. ATP Hydrolysis Activity Assays

A multiple time point activity assay was used to quantify Spa47 activity (ATP hydrolysis rates) from collected size exclusion chromatography (SEC) fractions of each of the Spa47 variants. The reactions were initiated by combining Spa47 protein with a prepared ATP solution resulting in a final concentration of 0.5 μM Spa47 (monomer or oligomer), 1 mM ATP, 10 mM MgCl_2_, and 0.5 μCi (∼300 nM) α-^32^P-ATP. Reaction aliquots were removed and rapidly quenched with 0.25 M EDTA 60, 120, 300, 900, and 1800 s following reaction initiation. Cleaved α-^32^P-ADP and uncleaved α-^32^P-ATP were separated for each time point via TLC and quantified following exposure to a phosphor imaging screen and detection using a Storm 860 molecular imager [53].

### 4.5. Far-UV Circular Dichroism (CD)

Far-UV CD spectra and thermal stability profiles were collected for SEC-isolated monomeric and oligomeric fractions of each Spa47 mutant. CD data were obtained using a JASCO model J-1500 spectropolarimeter with a Peltier temperature controller (Jasco, Easton, MD, USA). Samples were placed in 0.1 cm path length quartz cuvettes and equilibrated to 10 °C prior to collecting spectral data from 200 to 260 nm. Collection parameters included a 50 nm min^−1^ scan rate, 1 s data integration, and 0.5 nm spectral resolution. Thermal stability profiles were obtained by collecting CD data at 222 nm as the sample temperature was increased from 10 °C to 90 °C at 0.3 °C/min. Protein secondary structure thermal transition temperatures (Tm) were obtained by determining the minimum of the first derivative curve of the sigmoidal function fit to the variable temperature CD data. All CD analyses were performed at 0.5 mg/mL protein concentration and values presented as the mean ± S.D. of three independent measurements. All CD signals were converted to mean residue molar ellipticity.

### 4.6. Shigella-Induced Erythrocyte Hemolysis

The impact of the Spa47 tyrosine mutations on type three secretion system-mediated red blood cell hemolysis was investigated using a previously published protocol [54]. Briefly, *Shigella* strains expressing the engineered Spa47 tyrosine mutants as well as control strains including a *spa47* knockout, a *spa47* knockout vector control containing the pWPsf4 expression vector, but no gene for Spa47, and a *spa47* knockout strain complemented with wild-type *spa47* were grown in tryptic soy broth containing appropriate antibiotics. The cultures were grown to an OD600 of 1.0 prior to pelleting via centrifugation. The bacteria were resuspended in PBS and each *Shigella* suspension was introduced to ∼5 × 10^8^ red blood cells in a 96-well microtiter plate. Centrifugation at 2300× *g* initiated contact between the red blood cells and the *Shigella* prior to a 1 h incubation at 37 °C to allow T3SS translocon insertion into the blood cell membranes. The red blood cells and *Shigella* were then resuspended following the addition of 100 μL of cold PBS per well. The resuspended mix was again centrifuged to pellet the bacteria and erythrocytes. The soluble hemoglobin that was released as a result of membrane disruption by the *Shigella* was quantified by measuring absorbance at 545 nm and compared with the levels resulting from *S. flexneri* expressing wild-type Spa47 and a PBS negative control solution lacking bacteria.

### 4.7. Bacterial Invasion of Epithelial Cells

*S. flexneri* invasion of cultured HeLa cells was monitored by a gentamicin protection assay using a modified version of a previously described protocol [55]. Briefly, HeLa cells were grown overnight in sterile 24-well plates containing DMEM supplemented with streptomycin, penicillin, and 10% fetal calf serum in an incubator maintaining and environment of 37 °C, 100% relative humidity, and 5% CO_2_. The engineered *Shigella* strains used throughout this study were grown in tryptic soy broth media at 37 °C and 200 RPM to an OD600 of 0.4 prior to introduction of equivalent bacterial loads to the cultured HeLa cells. Centrifuging the plates at 1000× *g* synchronized contact between the HeLa cells and *Shigella* and initiated invasion that was further supported by incubation at 37 °C for 30 min. The HeLa cells were then rinsed to remove most of the extracellular bacteria. *Shigella* that had not successfully invaded the HeLa cells were then killed by treatment with 50 μg/mL gentamicin. Bacteria that had invaded were visualized by lysing the host cells with 1% agarose in water and overlaying with a 2× LB agar solution. Overnight incubation at 37 °C resulted in colony formation from the individual *Shigella* released from the HeLa cells’ cytoplasm. Enumerating the resulting colonies provides a quantitative measure of the ability of the *S. flexneri* strains to invade host cells and initiate infection.

### 4.8. Quantitation of S. flexneri T3SS Translocator Secretion

Congo Red is a small diazo dye that effectively initiates secretion of T3SS translocator proteins from *Shigella* by triggering the natural response resulting from interaction with host cell membrane components [56]. Thus, treatment with Congo Red has proven to be a valuable tool capable of dissecting the impact of individual protein mutations, such as the Spa47 tyrosine phosphomimetics used in this study, on *Shigella* T3SS activation and protein secretion. The Congo Red secretion protocol has been described in detail elsewhere [57]. The *Shigella* strains described previously in this study were grown to an OD600 of 1.0 at 37 °C in tryptic soy broth containing appropriate antibiotics to maintain the transformed protein expression plasmid. The cultures were then rapidly chilled on ice to temporarily slow translocator protein expression and secretion. The bacteria were isolated by centrifuging the cultures and rinsing the resulting cell pellets to ensure removal of any proteins that had been secreted up to this point. The bacteria were then incubated at 37 °C for 15 min in sodium phosphate buffer containing 0.28 mg/mL Congo Red to activate the T3SS and initiate effector secretion. The *Shigella* were again cooled on ice to limit further secretion and were centrifuged at 13,000× *g* for 15 min to separate them from the supernatant which contained secreted T3SS effector proteins. The isolated proteins that were then separated by SDS-PAGE, transferred to PVDF membranes by Western blot, and visualized using anti-IpaC rabbit polyclonal antibodies and an Alexa 647 goat anti-rabbit secondary antibody. The rabbit polyclonal anti-IpaC antibodies have been previously validated for specific detection of IpaC in a similar Congo Red secretion assay [35]. Secreted IpaC levels were detected and compared using a Bio-Rad ChemiDoc imaging system and the associated Image Lab analysis software. Antibodies against the cytoplasmic glycolytic enzyme glyceraldehyde 3-phosphate dehydrogenase (GAPDH) served as a lysis control, verifying that IpaC detected in the supernatant resulted from T3SS activity.

## Figures and Tables

**Table 1:** Secondary Structure Thermal Stability of Engineered Spa47 Tyrosine Mutants.

Spa47	Melt Temperature (Tm) ^a^ (°C ± S.D.)	
	Monomer	Oligomer
Wild-Type Spa47	44.4 ± 0.2	43.5 ± 1.5
Spa47^Y274F^	42.1 ± 0.5 *	41.6 ± 0.1
Spa47^Y274A^	39.5 ± 0.4 *	41.8 ± 1.4
Spa47^Y274E^	40.1 ± 0.2 *	45.1 ± 0.5
Spa47^Y398F^	43.9 ± 0.5 *	44.1 ± 1.7
Spa47^Y398A^	41.3 ± 0.2 *	40.5 ± 1.9 *
Spa47^Y398E^	36.0 ± 0.6 *	N/A

^a^ Secondary structure thermal stability of engineered Spa47 mutants was quantified by monitoring the CD signal at 222 nm as the sample temperature was increased from 10 °C to 90 °C. All results are reported as the mean Tm ± S.D. from three independent analyses spanning two separate protein preparations. (*) Indicates statistical significance compared to the appropriate oligomer state of wild-type Spa47 protein (one-way ANOVA followed by a Dunnett’s post hoc test, *p* ≤ 0.05).

**Table 2:** Effect of Engineered Spa47 Phosphomimetic Mutations on *Shigella* Hemolysis and Invasion Phenotype.

Spa47	Relative Phenotype (% ± S.D.)	
	Hemolysis ^a^	Invasion ^b^
Spa47 null	3 ± 0 *	1 ± 1 *
Vector Control	3 ± 0 *	0 ± 0 *
Wild-Type Spa47	100 ± 0	100 ± 0
Spa47^Y274F^	96 ± 7	99 ± 13
Spa47^Y274A^	78 ± 8 *	51 ± 9 *
Spa47^Y274E^	46 ± 3 *	32 ± 11 *
Spa47^Y398F^	119 ± 3 *	90 ± 10
Spa47^Y398A^	132 ± 6 *	110 ± 20
Spa47^Y398E^	117 ± 8 *	119 ± 16

^a^ Hemolysis results are presented as a percentage of the hemoglobin released from red blood cells by the *S. flexneri* strain expressing wild-type Spa47. ^b^ Invasion results are presented as a percentage of the number of colonies resulting from the *S. flexneri* strain expressing wild-type Spa47. All data are presented as the mean ± S.D. resulting from three independent experimental data sets spanning two biological replicates. (*) Indicates statistical significance compared to the wild-type Spa47 strain (one-way ANOVA followed by a Dunnett’s post hoc test, *p* ≤ 0.05).

## Data Availability

Not applicable.

## Supplementary Materials

The following are available online at https://www.mdpi.com/article/10.3390/pathogens11020202/s1, Figure S1: Circular Dichroism characterization of phosphomimetic Spa47 mutants.
