# Supplementary material for: Phosphomimetic Tyrosine Mutations in Spa47 Inhibit Type Three Secretion ATPase Activity and Shigella Virulence Phenotype
Source: Pathogens. 2022 Feb 3;11(2):202. doi: 10.3390/pathogens11020202 (PMC8876561; doi:10.3390/pathogens11020202)
Supplement: Supplementary file 1 [file pathogens-11-00202-s001.zip › pathogens-1566396-supplementary.pdf]

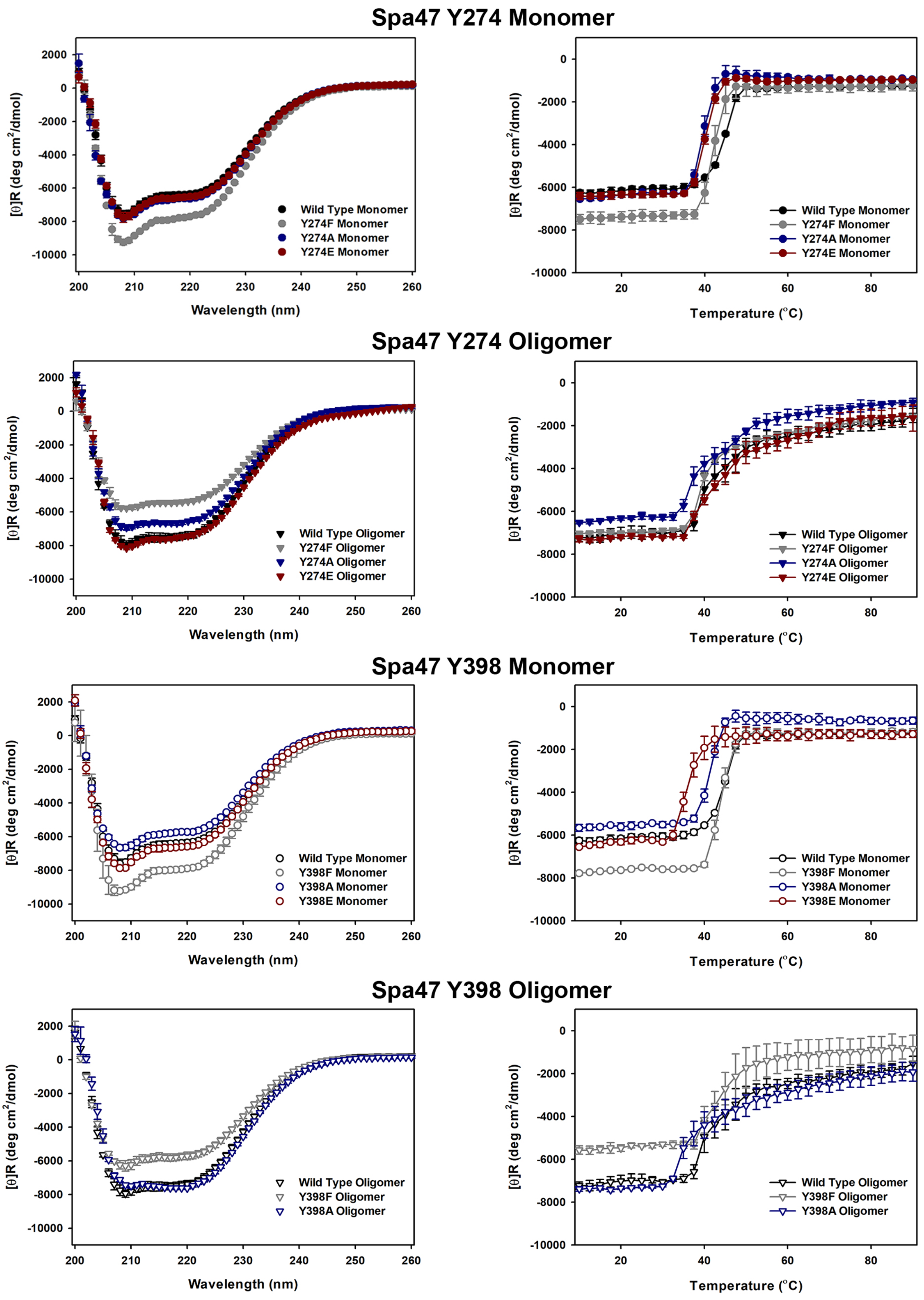

Supplementary Figure S1. Circular dichroism (CD) analysis of engineered Spa47 tyrosine mutants. Far-UV CD spectra of wild-type and mutant Spa47 oligomers and monomers contain minima at 208 nm and 222 nm, all consistent with highly α-helical secondary structure content (Left). Thermal unfolding profiles were collected by monitoring CD signal at 222 nm while sample temperatures were increased from 10 C to 90 C (Right). All CD experiments were performed in triplicate and data are presented as mean ± S.D.
